# Proof-of-concept study of a CD-type microfluidic titration system with an ISFET sensor for microlitre-scale analysis

**DOI:** 10.1007/s00604-025-07574-3

**Published:** 2025-10-02

**Authors:** Juntao Yang, Shoji Yamamoto, Kazuhiro Morioka, Akihide Hemmi, Hajime Kayanne, Hizuru Nakajima

**Affiliations:** 1 Department of Applied Chemistry, Graduate School of Urban Environmental Sciences, Tokyo Metropolitan University, 1-1 Minami-Osawa, Hachioji, Tokyo, 192-0397 Japan; 2 Department of Biomedical Analysis, School of Pharmacy, Tokyo University of Pharmacy and Life Sciences, 1432-1 Horinouchi, Hachioji, Tokyo, 192-0392 Japan; 3 Mebius Advanced Technology Ltd., 3-31-6 Nishiogi-Kita, Suginami-Ku, Tokyo, 167-0042 Japan; 4 Department of Earth and Planetary Science, The University of Tokyo, 7-3-1 Hongo, Bunkyo-Ku, Tokyo, 113-0033 Japan; 5 Department of Civil Engineering, The University of Tokyo, 7-3-1 Hongo, Bunkyo-Ku, Tokyo, 113-8656 Japan

**Keywords:** Microfluidics, ISFET sensor, Acid‒base titration, Centrifugal lab-on-a-disc, pH monitoring

## Abstract

Conventional titration methods rely on bulky equipment, large sample volumes, and manual reagent handling, limiting their integration into portable and microlitre-scale analytical platforms. This constraint persists despite the growing demand for compact chemical quantification tools in decentralized diagnostics and environmental monitoring. A key challenge remains the lack of precise, automated titration systems that can operate without external pumps, fragile electrodes, or complex tubing. To address this gap, we present a CD-type centrifugal microfluidic device integrated with an ion-sensitive field-effect transistor (ISFET) sensor for automated acid–base titration in microlitre-scale samples. Rotation-driven fluid control is employed in the lab-on-a-disc system, enabling sequential acid addition without external pumps or valves. The 3D-printed microfluidic chip incorporates ten reagent reservoirs connected to an outer reaction chamber, allowing precise titrant delivery through controlled rotational speeds. The ISFET sensor, featuring a Ta₂O₅ sensing membrane and a carbon-based reference electrode, exhibited high sensitivity (51.3 mV/pH) and notable linearity (*R*^2^ = 0.9929) across a broad pH range. Titration experiments using 300 μL of a sodium carbonate solution and 0.1 mol/L hydrochloric acid successfully revealed equivalence points associated with carbonate neutralization, closely matching the results obtained with a commercial pH meter. These findings validate the measurement reliability and reproducibility of the device and demonstrate its potential as a portable and low-cost titration platform. The proposed approach advances the miniaturization and automation of titration methods, offering promising applications in field-deployable chemical analysis, marine biogeochemistry, and point-of-care diagnostics.

**Graphical Abstract:**

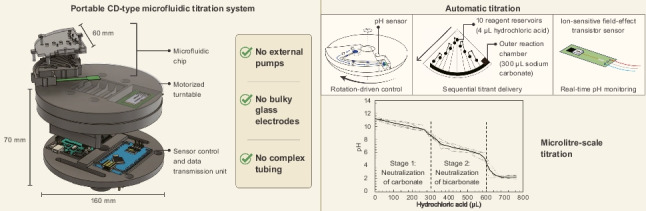

## Introduction

The increasing demand for portable and miniaturized analytical systems has driven research into compact and efficient chemical quantification techniques suitable for field-based and point-of-care applications. These platforms are essential for addressing emerging challenges in decentralized environmental monitoring, rapid clinical diagnostics, and microscale chemical synthesis. However, no existing system has yet combined discrete microlitre-scale titrant delivery with in situ, high-precision pH monitoring in a truly portable format. Addressing this gap is the central objective of this study, in which we present a centrifugal microfluidic titration system integrated with ISFET-based pH sensing.

Among the key analytical methods, titration is one of the fundamental techniques in chemical analysis and is widely employed for the precise quantification of chemical species in various fields, including environmental monitoring, clinical diagnostics, and chemical manufacturing. Conventional titration systems provide high reliability and accuracy; however, such systems generally require relatively large sample volumes (typically tens of millilitres or more), manual reagent handling, and benchtop instrumentation [1]. In particular, conventional systems often rely on bulky glass electrodes that are fragile and require internal liquid junctions, making them unsuitable for miniaturized platforms. Furthermore, manual titration procedures typically involve continuous reagent delivery and visual endpoint determination, both of which present challenges for automation and integration into compact devices. These limitations pose significant challenges, particularly in applications requiring precise analysis at the microlitre scale, such as biological fluids, microchemical synthesis, and localized environmental monitoring. In these cases, conventional titration methods may suffer from low sample efficiency, labour-intensive operation, and the inability to be deployed in portable or onsite analytical settings. Therefore, the development of miniaturized and automated titration systems has become increasingly necessary [2].

Various microfluidic approaches have been explored to achieve automated titration systems. For example, paper-based microfluidic titration systems offer a simple structure and low-cost implementation, demonstrating the feasibility of rapid semiquantitative analysis of vitamin C [3]. However, their imprecise mixing of reagents and reliance on visual colour change to determine titration endpoints limit quantitative accuracy, making it difficult to generate reproducible titration curves or detect subtle concentration differences. Similarly, thread-based microfluidic analytical devices (µTADs) involve the use of precoated reagents on threads for acid‒base titration, thereby quantifying concentrations based on the colour-change distance [4]. Although promising, these systems rely on colorimetric detection, which is susceptible to variations in environmental lighting conditions and sample matrix interference. Furthermore, in continuous-flow microfluidic titration systems developed for educational purposes, mathematical models are used to predict and control titration reactions involving strong acids and bases, weak acids and bases, and polyprotic acids, achieving high reproducibility [5]. Despite their advantages, such systems require external pumps and tubing, which complicates their integration into portable analytical platforms.

Recent advances have also expanded the scope of miniaturized sensing and automation. Paper-based analytical devices coupled with digital colorimetry have enabled microscale titration of weak acids with improved quantitation [6]. Centrifugal microfluidic platforms have progressed from digital valving control [7] to fully integrated, automated workflows for point-of-care diagnostics [8]. In parallel, the integration of ion-sensitive electrodes into microfluidic chips has been demonstrated for pH monitoring [9], and ion-selective potentiometric microsensors have been applied to nutrient analysis in soil environments [10]. These developments highlight the rapid progress in portable electrochemical sensing and lab-on-a-disc automation.

Despite ongoing advancements in the development of microfluidic titration systems, several core challenges remain unaddressed. These studies represent significant advancements in the miniaturization and simplification of titration techniques. However, real-time, high-precision pH monitoring systems remain limited. Although paper- and thread-based systems are suitable for simple quantification, they still face challenges in terms of obtaining precise titration curves and enabling stepwise reagent delivery. Moreover, precise control of reagent addition and real-time detection in small-volume titration remain technical challenges, particularly in fully integrated microfluidic systems [11, 12]. Accurate in situ pH monitoring is critical for acid–base titration, yet conventional glass electrodes are poorly suited for miniaturized or embedded applications owing to their size, fragility, and need for internal liquid junctions.

To overcome these challenges, centrifugal microfluidic platforms—also referred to as lab-on-a-disc systems—have attracted considerable attention. In these systems, rotation-induced centrifugal force is used for fluid manipulation, thus eliminating the need for external pumps or valves, reducing the risk of contamination, and enabling precise, sequential liquid handling in a compact and cost-effective format [13–16]. This modularity allows various functional components—such as reagent reservoirs, mixing zones, and detection chambers—to be integrated onto a single disc and arranged flexibly according to analytical needs. As a result, centrifugal microfluidic systems offer scalable and versatile workflows that are ideally suited for developing automated titration platforms.

Although various microfluidic platforms have been proposed for miniaturized titration, few systems have achieved integrated reagent delivery and in situ pH detection at microlitre resolution without reliance on bulky instrumentation. Therefore, the objective of this study is to develop a compact, centrifugal microfluidic titration system that integrates ion-sensitive field-effect transistor (ISFET)-based pH sensing for real-time, microlitre-scale analysis without the need for bulky instrumentation. The proposed system comprises a 3D-printed CD-type microfluidic chip equipped with multiple reagent reservoirs and an outer reaction chamber. By controlling the rotational speed, reagents are sequentially transported without the need for external valves or actuators, thus enabling stepwise titration [17]. Additionally, the incorporation of an ISFET sensor with a Ta₂O₅ membrane and a carbon-based reference electrode facilitates high-sensitivity and stable real-time pH monitoring [18–20].

To evaluate the analytical performance of the proposed system, sodium carbonate—a model alkaline compound with well-characterized acid–base behaviour and environmental relevance—was titrated with hydrochloric acid in microlitre-scale experiments, enabling an assessment of system accuracy and precision. Notably, a 300-μL sample was titrated with stepwise additions of 4 μL, yielding a well-defined titration curve. The results demonstrated excellent agreement with those obtained with a commercial pH metre, confirming that the use of a CD-type microfluidic platform integrated with ISFET sensors is a promising approach for miniaturized, low-cost, and high-precision microlitre-scale titration. These findings indicate the potential for broader adoption of ISFET-integrated microfluidic titration systems in clinical diagnostics, environmental monitoring, and onsite chemical analysis, where conventional approaches remain impractical due to volume, portability, or cost constraints. By demonstrating precise titration at the microlitre scale using low-cost and scalable components, this study contributes to the advancement of compact analytical platforms for decentralized and resource-limited settings.

## Experimental

### Materials and reagents

All the chemicals used in this study were of analytical grade. Sodium carbonate (Na₂CO₃, anhydrous, purity ≥ 99.8%, special grade; Kanto Chemical Co., Inc., Tokyo, Japan) was used as received without further drying. A hydrochloric acid volumetric standard solution (c(HCl) = 0.1000 mol L⁻^1^ at 20 °C; certified range 0.0995–0.1005 mol L⁻^1^; expanded uncertainty *U* =  ± 0.0003 mol L⁻^1^, *k* = 2, 95% confidence; Merck KGaA, Darmstadt, Germany, Certipur) was used for titration experiments. Standard buffer solutions with pH values of 1.68 (oxalate), 4.01 (phthalate), 6.86 (phosphate), 9.18 (borate), and 10.01 (carbonate) were used as received without dilution, equilibrated to 25 °C prior to use, and stored at 25 ± 1 °C in the dark in airtight; these buffer solutions were obtained from FUJIFILM Wako Pure Chemical Corporation (Osaka, Japan) and employed to evaluate the response of the pH sensor. Ultrapure water (18.2 MΩ·cm; Milli-Q system, Merck Millipore) was used for all solution preparation and rinsing. All the solutions were handled in capped vessels to minimize CO₂ uptake. A laboratory-prepared sodium carbonate solution (pH approximately 11.2) was used as the analyte for the titration tests. The solution was prepared by dissolving 1.06 g of anhydrous sodium carbonate (Na₂CO₃) in distilled water and diluting the solution to 100 mL in a volumetric flask. The solid sample was weighed using an analytical balance (GR-120, A&D Company, Tokyo, Japan).

The ISFET sensor employed in this study was based on a silicon substrate with a sputtered tantalum pentoxide (Ta₂O₅) film as the pH-sensitive membrane. A commercial FET (model 2SK208-Y; Toshiba Electronic Devices and Storage Co.) and silver/silver chloride paste (60/40; product code 901,773-50G; Sigma‒Aldrich) were applied for sensor assembly. Five-millimetre-wide carbon fibre tape (HTS40; Sano Factory) and marine-grade epoxy resin (Loctite; Henkel Corp.) were employed to construct the reference electrode [21]. The microfluidic chip was fabricated using a stereolithography-based 3D printer (FlashForge Foto 8.9 s) and a transparent photopolymer resin (LCD Standard Resin; FlashForge).

The microfluidic chip was printed from a transparent methacrylate-based SLA resin, chosen for its rigidity, print fidelity, and sufficient optical clarity to visually confirm liquid movement. In our experiments with aqueous buffers, 0.1 M HCl, and carbonate solutions, no visible swelling, leakage, or degradation of the resin was observed over repeated cycles. SLA resins are known to be stable in water and mild acids; to further minimize leachables that could interfere with electrochemical sensing, the printed parts were twice cleaned in isopropanol and subjected to UV postcuring. ISFET readouts showed no drift or artefacts attributable to the substrate material. The reservoirs were sealed with high-tack acrylic tape, which provided leak-tight and reproducible bonding; no delamination occurred under the rotational conditions tested. For extended or more aggressive chemistries, stronger bonding methods (UV-curable adhesives and thermal fusion) can be adopted.

### Device fabrication

**Fig. 1:**
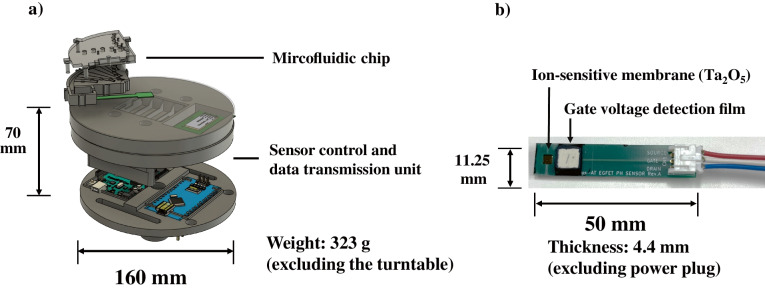
**a** Schematic representation of the developed CD-type titration device, which comprises a microfluidic chip (top) and a sensor control and data transmission module (bottom). The housing was fabricated using a stereolithography-based 3D printer. **b** Photograph of the ISFET sensor adopted in this study, comprising a Ta₂O₅ ion-sensitive membrane and a gate voltage detection film mounted on a commercial FET element

The configuration and external appearance of the titration device developed in this study are shown in Fig. 1. The device comprises a CD-type microfluidic chip and a sensor control and data transmission unit that processes and records signals from an ISFET-based pH sensor. The main body measures 160 mm in diameter and 70 mm in height, with a total weight of approximately 323 g excluding the turntable (Fig. 1a). The ISFET sensor incorporates a pH-sensitive membrane made of tantalum pentoxide (Ta₂O₅) in the gate region (Fig. 1b). Changes in the gate voltage are detected by the control unit and transmitted wirelessly to a PC via Bluetooth communication. The fabrication processes of each component are described in the following subsections.

#### Fabrication of the CD-type microfluidic chip

The CD-type microfluidic chip developed in this study was designed to use centrifugal force for fluid transport, thus enabling pump- and valve-free operation conditions. This structure is expected to facilitate simplification, miniaturization, and low-power operation of the system, which are desirable features for future field applications and portable analytical platforms. In addition, the system was designed to achieve titration in microlitre-scale volumes, which reduces reagent consumption and sample requirements. The chip integrates multiple liquid reservoirs, microchannels, a reaction chamber, and air vents on a single substrate. By incrementally controlling the rotation speed, reagents can be sequentially delivered and mixed within the chip.

**Fig. 2:**
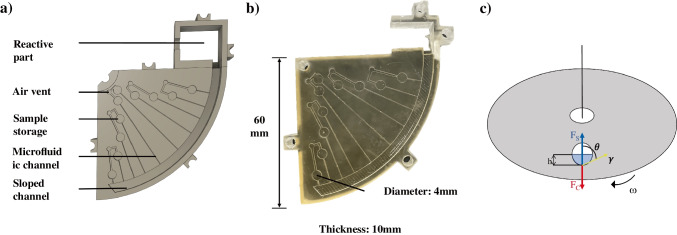
**a** Structural design of the CD-type microfluidic chip, illustrating the reservoirs, microchannels, sloped channel, air vents, and outer reaction chamber. **b** Photograph of the fabricated CD-type microfluidic chip. **c** Schematic representation of the balance between the centrifugal (*F*_c_) and capillary (*F*_s_) forces acting on the liquid within the rotating microfluidic device

The structure and operating principle of the chip are shown in Fig. 2. As shown in Fig. 2a and Fig. 2b, ten liquid reservoirs are radially arranged and connected to the outer reaction chamber via microchannels. An air vent is located above the reaction chamber to stabilize the pressure and ensure smooth liquid delivery.

Fluid actuation within the chip is governed by the balance between the centrifugal force (*F*_c_) and the capillary retention force (*F*_s_), as shown in Fig. 2c. The centrifugal force acting on a liquid segment at radial distance *R* is defined as follows:

1 $$Fc=R{\omega }^{2}\rho {r}^{2}h$$

where $\omega$ is the angular velocity, *ρ* is the liquid density, *r* is the outlet width, and *h* is the fluid height [12]. The capillary force that opposes fluid release is due primarily to surface tension and can be expressed as follows:

2 $$Fs=4r\gamma \text{cos}\theta$$

where *γ* is the surface tension and *θ* is the contact angle. Assuming that *F*_c_ = *F*_s_, the critical rotational speed at which the liquid begins to flow can be estimated as follows:

3 $$f=\frac{60}{2\pi }\sqrt{\frac{2\gamma \left|\left.\text{cos}\theta \right|\right.}{R\rho rh}}$$

The liquid properties and wetting parameters (25 °C) were as follows: the density was *ρ* = 1000 kg/m^3^, the static contact angle on the printed resin was *θ* = 29.6° (observed on coupons with the same surface treatment), and the surface tension was* γ* = 0.0701 N/m, which was obtained from a Young–Laplace plus hydrostatic equilibrium measurement. In a square microchannel of side *r* = 250 µm, the difference in the equilibrium level relative to an external free surface was *H* = 0.275 mm. Viscous losses were neglected; thus, the estimate acted as a modest upper bound for the release speed. The resulting theoretical values were used to benchmark the experimental thresholds reported below.

By tuning these physical parameters, the chip enables each reservoir to release fluid only when the rotational speed exceeds its specific threshold. This mechanism facilitates stepwise reagent delivery from multiple reservoirs by simply increasing the rotational speed [12].

The chip design includes ten liquid reservoirs (4-mm diameter, 0.5-mm depth), positioned at radial distances ranging from 15 to 51 mm from the rotational centre in 4-mm increments, each connected via microchannels (250-µm width, 250-µm depth) to an outer reaction chamber with a total volume of approximately 1100 μL. The system was tuned to provide sequential fluid release at rotational speeds ranging from 340 to 900 RPM.

The chip was fabricated using a stereolithography-based 3D printer (FlashForge Foto 8.9 s) with a transparent LCD-compatible photopolymer resin (Standard Resin, Natural colour, FlashForge). After printing, the chip was immersed in 2-propanol (99.5%) and ultrasonically cleaned for 10 min to remove any residual uncured resin. The rinsed chip was then dried with nitrogen and cured under ultraviolet light (UV-LED LIGHT SOURCE Area Type H-20AH4 2) at 405 nm with an intensity of approximately 320 mW/cm^2^ for 10 min. This postprocessing step enhanced the mechanical strength, structural integrity, and chemical resistance of the chip, ensuring its suitability for subsequent fluidic operations and pH monitoring.

In this proof-of-concept study, only a 90° sector of the disc was populated with ten reservoirs. We adopted this sector-based layout for two practical reasons. First, the build envelope of the SLA printer does not accommodate single-shot fabrication of a full disc of this geometry; splitting the disc into multiple wedges would introduce seams and cumulative alignment/tolerance errors in microchannels and valving features, undermining quantitative comparability across runs. Second, because the study relies on frequent, experiment-driven revisions, fabricating a partial disc markedly reduced trial-and-error cost (shorter print times and less material), enabling rapid design–test–revise cycles. As an added benefit, the sector format allowed for straightforward optical access and on-disc ISFET wiring during the calibration and comparison experiments. This constraint reflects a deliberate scope limit for the prototype; the geometry is readily expanded to 360° or stackable into multilayer (3D) formats in future iterations.

#### Fabrication of the ISFET electrode and the reference electrode

The ISFET and reference electrodes used in this study were custom-fabricated to enable stable pH sensing in the microfluidic titration system. The ISFET electrode comprises a silicon substrate with a pH-sensitive tantalum pentoxide (Ta_2_O_5_) membrane and a discrete FET element (2SK208-Y, Toshiba). The Ta_2_O_5_ layer was sputtered for 240 s via a CFS-4EP-LL sputtering system (Shibaura Mechatronics) installed in the Takeda Advanced Science Cleanroom at the University of Tokyo. The film thickness (approximately 37.3 nm) was measured using an optical interferometric film thickness measurement system (TohoSpec 3100, Toho Technology). The uncoated side of the silicon substrate was electrically connected to a printed circuit board (PCB) using silver/silver chloride paste (60/40; product code 901,773-50G; Sigma‒Aldrich), followed by heat curing at 150 °C for 30 min in a temperature-controlled oven (ST-110, ESPEC). To ensure electrical insulation, all nonsensing areas were coated with black epoxy resin (INPEI BLACK, Sunhayato Co., Ltd.) prepared at a 2:1 base-to-curing agent ratio. The FET element was soldered onto the PCB, and its gate terminal was connected to the Ta_2_O_5_-coated sensing area via a conductive wire. The fabricated ISFET sensor occupies an area of approximately 5 × 5 mm on the silicon substrate. The entire assembly, including the epoxy resin insulation, measures approximately 50 × 11.25 × 4.4 mm.

The reference electrode was fabricated using 5-mm-wide carbon fibre tape (HTS40, Mitsubishi Chemical Corp.) as the gate-voltage reference. A 3–5-cm segment of carbon fibre tape was vertically inserted into a silicone rubber mould with a cross-shaped cut, and a rubber sheet (1 × 1 cm) with a 5 × 5 mm window was placed above it for alignment. Marine-grade epoxy resin (Loctite, Henkel) was poured into the mould to encapsulate the carbon fibres, which were subsequently cured at room temperature for 24 h to ensure waterproofing and mechanical stability. After curing, the mould was removed, and the excess resin was trimmed to expose the cross section of the carbon tape. The electrode was mounted onto the PCB with carbon paste, heat-cured at a temperature of 150 °C for 30 min, and insulated with additional epoxy resin to prevent electrical interference. Only the top surface of the carbon fibre remains exposed to the solution during the measurement.

The fabricated ISFET sensor was functionally tested using standard buffer solutions (pH values of 1.68, 4.01, 6.86, 9.18, and 10.01). These solutions were chosen to cover a broad range of pH values spanning acidic, neutral, and basic conditions, which enables a comprehensive evaluation of the sensor’s voltage response across physiochemically relevant domains for environmental and clinical applications. Furthermore, titration was performed using a sodium carbonate solution, with parallel measurements obtained from the ISFET sensor and a commercial glass pH electrode (Horiba F-70), to assess the sensor’s performance under dynamic acid‒base conditions.

### Evaluation of the microfluidic performance

The CD-type microfluidic chip was designed to enable stepwise delivery of reagents using centrifugal force. In this section, we describe the experimental procedure adopted to evaluate the fluidic control performance of the chip by determining the critical rotational speed at which liquid is released from each reservoir.

In the evaluation experiment, 4 μL of black-dyed distilled water (using Kyodai Food HM Food pigment) was loaded into the ten reagent reservoirs to allow visual observation of fluid movement. To prevent unintended spillage during rotation, the reservoirs were sealed by covering the entire top surface of the CD with strong double-sided adhesive tape (ad006, VAN-SONIC) after loading. The assembled device was mounted on a motorized rotational platform, and the rotational speed was increased stepwise from 200 to 1000 RPM in increments of 13 RPM. At each rotational speed, whether the liquid had been released into the outer reaction chamber was visually confirmed. Afterwards, the critical rotational speed for fluid release from each reservoir was recorded. The relationship between the reservoir position (radial distance) and release threshold is presented in the “Results and discussion” section.

### Titration system configuration

The titration system developed in this study comprises a CD-type microfluidic chip, an ISFET pH sensor, a motorized turntable, and a Bluetooth-enabled data acquisition unit. This setup enables automatic titration and real-time pH monitoring of microlitre-scale samples without the need for external pumps or complex tubing.

The rotation mechanism was driven by a DC motor module (1-Q-EC Amplifier DEC Module 50/5) controlled by an Arduino MKR Zero microcontroller. The spin speed was regulated via pulse width modulation (PWM) using Arduino IDE software. The rotational speed was continuously monitored through a noncontact optical tachometer (RM-01U-M; MonotaRO Co., Ltd.), with reflective tape affixed to the outer edge of the disc. The system was powered by eight 1.2-V AA NiMH batteries (totalling 9.6 V), which supplied a stable voltage to the Arduino board, the ISFET preamplifier, and the HC-06 Bluetooth module. Real-time voltage readings from the ISFET sensor were transmitted wirelessly via Bluetooth and recorded on a PC using Tera Term 5 software. To ensure mechanical balance during rotation, the sensor and Bluetooth modules were positioned symmetrically on opposite sides of the disc—a configuration that was found to stabilize rotational dynamics and prevent wobbling during operation. The microfluidic chip was mounted at the centre of the disc, with the ISFET sensor inserted into the outer reaction chamber.

### Titration operation and data acquisition

In the simulated titration experiment, 300 μL of a 0.1 mol/L sodium carbonate solution was introduced into the outer reaction chamber, and each of the ten reagent reservoirs was preloaded with 4 μL of 0.1 mol/L hydrochloric acid. This configuration enabled stepwise acid addition with a constant titrant volume at each stage.

In this study, titration was performed by sequentially increasing the rotation speed of the turntable to match the critical rotational threshold of each reservoir. At each step, the target speed was maintained for 8 s to ensure complete delivery of the titrant, followed by a brief reduction to 150 RPM for 1 s to promote mixing. This release–mix–pause sequence was repeated three times at each stage to ensure completion of the acid–base reaction before proceeding to the next step. These operation protocols were subsequently applied in the “Titration with the ISFET sensor and the CD-type microfluidic device” section to evaluate the analytical performance of the integrated titration system under automated microlitre-scale conditions.

The ISFET sensor remained immersed in the reaction chamber throughout the titration process, and the changes in the gate voltage corresponding to the changes in pH were continuously monitored. The titration endpoint was identified via the first derivative method, in which the maximum dpH/dV value was adopted as the equivalence point—the stage at which stoichiometrically equivalent amounts of acid and base have reacted, resulting in a sharp change in the pH curve. The recorded signals were transmitted wirelessly via Bluetooth to a PC, allowing real-time tracking of the changes in pH throughout the titration process.

### Data analysis

Sensor sensitivity (in mV/pH) was determined from the slope of the linear regression line fitted to the sensor output voltage against the pH values of the standard buffer solutions. The correlation coefficient (*R*^2^) was calculated from the same regression analysis using the least-squares method. Reproducibility was assessed by calculating the relative standard deviation (RSD) from triplicate measurements at each pH point. All the statistical analyses were performed using Microsoft Excel.

## Results and discussion

### Characterization of the ISFET sensor

To evaluate the analytical performance of the proposed CD-type microfluidic titration system integrated with ISFET sensors, we first characterized the electrochemical response of the fabricated sensor to pH variations. Standard pH buffer solutions (pH 1.68, 4.01, 6.86, 9.18, and 10.01) were used to assess the sensitivity, linearity, and reproducibility of the sensor across a broad range of pH values relevant to acid–base titration applications.

**Fig. 3:**
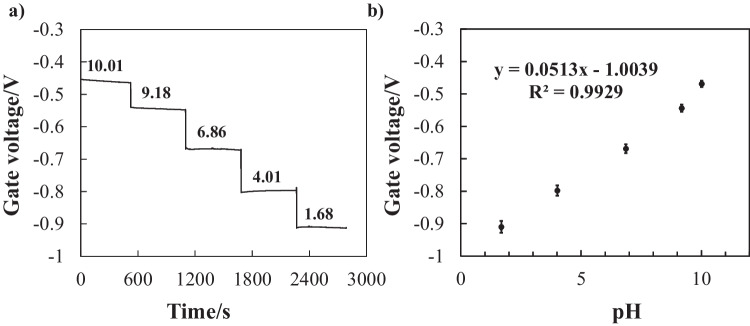
Response characteristics of the ISFET pH sensor. **a** Real-time gate voltage signals observed during immersion in various pH buffer solutions (pH: 10.01, 9.18, 6.86, 4.01, and 1.68). **b** Calibration curve showing the average gate voltage as a function of the pH. The error bars represent the standard deviation across three measurements for each buffer

The sensor responded rapidly and stably when immersed in each buffer solution, with the gate voltage stabilizing within a few seconds (Fig. 3a). For each pH condition, three independent measurements were obtained, and the average gate voltage was recorded. The calibration curve constructed from these measurements is shown in Fig. 3b.

Linear regression analysis yielded a sensitivity of 51.3 mV/pH and a correlation coefficient (*R*^2^) of 0.9929. These results are consistent with the theoretical Nernstian response of approximately 59.16 mV/pH at 25 °C for monovalent ion detection [22]. The relative standard deviation (RSD) values ranged from 0.1 to 0.8%, confirming satisfactory measurement reproducibility. Although the measured slope was slightly sub-Nernstian, such responses have been widely reported for Ta₂O₅-based ISFETs and generally attributed to a combination of surface-state effects and reference electrode stability [20, 23].

This calibration curve was applied in subsequent experiments to convert the gate voltage data into pH values. During calibration, transient noise was occasionally observed while the sensor was transferred between solutions, likely due to temporary loss of electrical contact between the gate and reference electrodes in air. However, the signal stabilized immediately upon immersion, and this effect was negligible under continuous measurement conditions.

### Evaluation of microfluidic flow control

Building upon the electrochemical characterization of the ISFET sensor, we next assessed the fluidic control properties of the CD-type microfluidic platform to ensure precise and sequential reagent delivery during titration. To ensure reliable and sequential delivery of the titrant in the CD-type microfluidic system, the flow control performance of each reservoir was evaluated by determining the minimum rotational speed needed to initiate fluid release. This evaluation was conducted using 4 μL of distilled water dyed with edible black colouring, which was loaded into each of the ten reservoirs on the fabricated microfluidic chip.

**Fig. 4:**
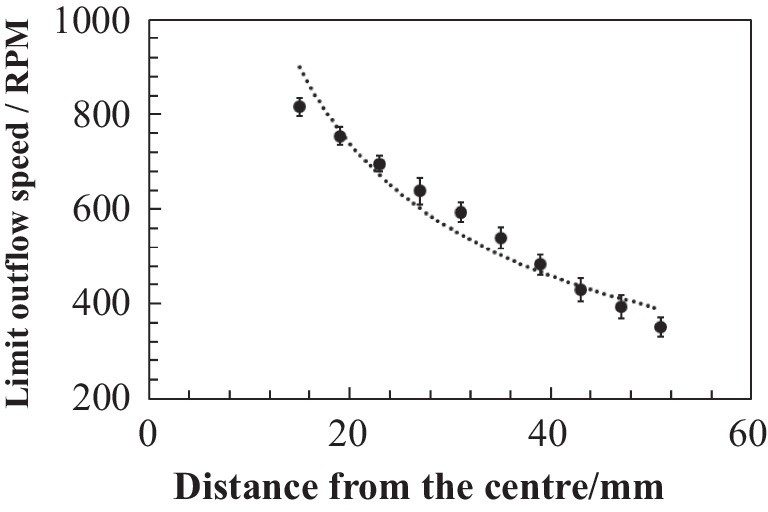
The threshold rotational speed decreases linearly with increasing reservoir radial position. Each data point represents the average of 15 measurements. The error bars indicate the standard deviation

The device was mounted on a motorized rotation stage and incrementally accelerated from 200 to 1000 RPM in 13-RPM steps. At each increment, visual observation was employed to determine whether fluid had exited a given reservoir and entered the downstream microchannel. The threshold rotational speed for flow initiation was recorded for each reservoir on the basis of 15 repeated measurements. The results revealed that each reservoir exhibited a distinct and reproducible threshold speed, which was influenced primarily by its radial position and the surface tension at the outlet. The measured thresholds ranged from approximately 340–900 RPM, allowing for a well-separated and predictable delivery sequence across all ten reservoirs. The relationship between the radial distance of each reservoir from the centre and its corresponding threshold rotational speed for liquid release is shown in Fig. 4. Each data point represents the average of repeated measurements with standard error bars. The dotted line represents the best-fit curve constrained to the theoretical exponent of − 0.5, with the equation *y* = 3132⋅*x*^−0.5^ and a coefficient of determination *R*^2^ = 0.9098. The fitted coefficient (3132) corresponds to an effective liquid head of approximately *h* ~ 4.6 mm when the other parameters (*γ*, *θ*, *ρ*, and *r*) are fixed. In contrast, geometric considerations of the reservoir dimensions suggest an effective head of *h* ~ 2.5 mm, which would yield a theoretical coefficient of 4218. Thus, the experimental value is smaller than the purely theoretical estimate. This discrepancy is most plausibly explained by dynamic wetting effects, whereby the advancing contact angle at release reduces |cosθ| relative to the static value used in the model, together with minor reductions in surface tension and dimensional tolerances of the outlet aperture *r*. These factors lower the capillary counterpressure (and hence the *F*_c_ = *F*_s_ threshold), causing release to occur at lower RPMs. A modest increase in the effective hydrostatic head *h* under rotation may further contribute to this behaviour. Importantly, no inversion of the release order was observed across reservoirs, and the actuation windows remained well separated, ensuring robust stepwise dosing.

### Titration comparison between the ISFET sensor and a commercial pH metre

To evaluate microfluidic flow control, we next compared the titration performance of the ISFET sensor against that of a conventional pH metre to assess its analytical accuracy and real-world applicability. To evaluate the performance of the fabricated ISFET pH sensor, a comparative titration experiment was conducted using a commercial glass electrode pH metre under identical conditions. A 0.1 mol/L sodium carbonate solution (10 mL) was titrated with 0.1 mol/L hydrochloric acid in 0.1-mL increments, and the resulting pH was measured with both a commercial pH metre (Horiba F-70) and the ISFET sensor developed in this study. The ISFET sensor and its reference electrode were immersed in the same solution, and the gate voltage was continuously recorded. Notably, the pH values were calculated based on the calibration curve described in the “Characterization of the ISFET sensor” section.

**Fig. 5:**
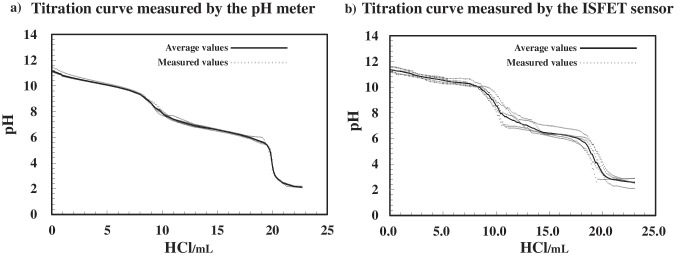
Comparison of the titration curves obtained with the ISFET sensor and a commercial pH metre for 10 mL of 0.1 mol L⁻^1^ Na₂CO₃ titrated with 0.1 mol L⁻^1^ HCl at 25 °C. **a** Titration curve obtained with a commercial pH metre. **b** Titration curve obtained with the ISFET sensor. The *x*-axis denotes the cumulative volume of HCl added from 0 mL. The curves are the means of five replicates; the markers indicate the first and second equivalence points

The titration curves obtained from the two sensors over five replicate experiments are shown in Fig. 5. Each curve clearly exhibits two inflection regions corresponding to the stepwise neutralization of carbonate and bicarbonate ions. The equivalence points determined with the commercial pH metre were 9.62 ± 0.18 mL (first point, pH 8.15 ± 0.12) and 19.96 ± 0.22 mL (second point, pH 4.20 ± 0.15, corresponding to an additional 10.34 ± 0.22 mL beyond the first equivalence point). In comparison, the ISFET sensor yielded equivalence points at 10.16 ± 0.50 mL (pH 8.36 ± 0.12) and 20 ± 0.31 mL (pH 4.15 ± 0.12, corresponding to an additional 9.84 ± 0.31 mL beyond the first equivalence point). Despite minor variations in the absolute titrant volume, the ISFET sensor-derived curves closely matched those of the commercial pH metre in terms of both the pH profile and inflection point structure. The observed differences—generally within ± 0.5 mL across five replicate trials—demonstrate consistent titration precision and confirm the ability of the ISFET sensor to provide accurate acid–base titration. These results demonstrate that the developed ISFET sensor offers comparable analytical performance to that of conventional glass electrodes while being more compact and integrable into microfluidic systems.

### Titration with the ISFET sensor and the CD-type microfluidic device

Titration experiments were performed using the CD-type microfluidic device described in the “Titration system configuration” section to evaluate its performance under automated, small-volume conditions. The goal was to determine whether the system could reproduce typical acid–base titration curves using microlitre-scale samples without external pumps or manual operation.

A 300-μL aliquot of a 0.1 mol/L sodium carbonate solution was introduced into the outer reaction chamber, and ten peripheral reservoirs were each preloaded with 4 μL of 0.1 mol/L hydrochloric acid. This process was repeated after each 40-μL sequence to facilitate continued titration, ultimately enabling a total titrant volume of approximately 800 μL.

Throughout the experiment, the ISFET sensor remained submerged in the reaction chamber, continuously monitoring changes in the gate voltage associated with changes in the pH. These voltage signals were transmitted wirelessly via Bluetooth to a PC and converted into pH values based on the calibration curve established in the “Characterization of the ISFET sensor” section.

**Fig. 6:**
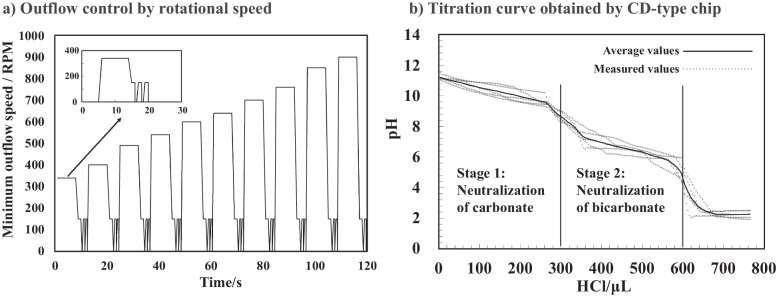
Operation profile and titration curves of the CD-type microfluidic device. **a** Programmed rotation-speed waveform for one titration step: the disc is driven to the reservoir-specific critical speed (set by its radial position), held for 8 s to fully release a 4 μL aliquot, and then dropped to 150 RPM for 1 s to promote mixing. This “release 8 s–mix 1 s” pulse is repeated three times (approximately 27 s per step) before advancing to the next step. **b** Titration curves of 0.1 mol/L sodium carbonate via the CD-type microfluidic device

As illustrated in Fig. 6a, each step used a standardized waveform: the disc was driven to the reservoir-specific critical speed, held for 8 s to discharge a 4 μL aliquot, and then dropped to 150 RPM for 1 s to promote mixing. This release–mix pulse was repeated three times (approximately 27 s per step) before advancing to the next reservoir. Across the sequence, the critical speed values spanned approximately 340–900 RPM.

The titration curves obtained from five independent trials are shown in Fig. 6b. Each curve exhibited two clear equivalence points corresponding to the stepwise neutralization of carbonate and bicarbonate ions. The first equivalence point was observed at an average pH of 8.45 ± 0.16, with a mean acid consumption of 307 ± 10 μL. The second equivalence point occurred at an average pH of 4.33 ± 0.23, with a mean consumption of 607 ± 13 μL. The variation in the titrant volume remained within ± 4%, confirming the ability of the system to achieve reliable and reproducible titrations with discrete 4-μL steps. These results validate the effectiveness of the ISFET-integrated CD microfluidic platform for small-volume, automated acid–base titration. Its demonstrated precision and reproducibility suggest strong potential for integration into compact analytical tools for decentralized chemical analysis, including environmental monitoring, clinical diagnostics, and portable onsite assays in resource-limited settings.

### Quantitative performance evaluation

To quantitatively evaluate the performance of the CD-type titration system developed in this study, the titration curves were compared among three instruments: (1) a commercial glass electrode pH metre, (2) a custom-fabricated ISFET sensor, and (3) a CD-type microfluidic device integrating the ISFET sensor. In all the cases, a 0.1 mol/L sodium carbonate aqueous solution was employed as the analyte, and 0.1 mol/L hydrochloric acid served as the titrant.

In conventional bulk titration experiments using 10-mL samples, a commercial pH metre detected the first and second equivalence points at 9.62 ± 0.18 mL (pH 8.15 ± 0.12) and 19.96 ± 0.22 mL (pH 4.20 ± 0.15), respectively. The developed ISFET sensor yielded equivalence points of 10.16 ± 0.50 mL (pH 8.36 ± 0.12) and 20 ± 0.31 mL (pH 4.15 ± 0.12), respectively. These results show a high level of agreement between the two sensors, with deviations in the titrant volume limited to approximately ± 0.5 mL (relative uncertainty: approximately 5%) and pH values within ± 0.2. This confirms the ability of the ISFET sensor to reliably track acid–base neutralization in a bulk-scale setting.

Titration was subsequently performed using a CD-type microfluidic device with a sample volume of 300 μL. Across five trials, the first equivalence point was observed at 307 ± 10 μL (pH 8.45 ± 0.16), and the second equivalence point was observed at 607 ± 13 μL (pH 4.33 ± 0.23). The observed variations in the titrant volume remained within ± 5%, which is consistent with the 4-μL dispensing resolution of the system. Ideally, 300 μL of titrant is theoretically needed to reach each of the two equivalence points, assuming complete neutralization of carbonate and bicarbonate ions. The present results fall within this expected range, supporting the reliability of volume control and reaction completeness. These findings indicate that even under miniaturized and automated conditions, the system successfully reproduced the expected titration profile with high consistency.

Overall, the CD-type system demonstrated a quantitative performance comparable to those of standard titration methods, despite operating with approximately 1/30 of the sample volume. The consistency of the equivalence points and pH transitions across all conditions underscores the reliability and suitability of the system for compact, field-deployable chemical analysis. These results highlight the broader applicability of the proposed system across decentralized diagnostic testing, microchemical process monitoring, and modular lab-on-disc devices. Its operational simplicity, precision, and scalability suggest promising directions for future integration into multiple-analyte sensing platforms or autonomous environmental assessment kits.

### Challenges and future prospects

This study confirmed the fundamental performance of an automated titration system integrating a centrifugal microfluidic chip and an ISFET sensor. However, several challenges that must be addressed in future improvements were also identified.

First, the current system operates with discrete titration steps of 4 μL, which imposes a limitation on the resolution compared with conventional continuous titration methods. This constraint may affect the detection accuracy of subtle pH changes, particularly in the steep regions of the titration curves. In the present prototype, only one-quarter (90°) of the CD chip area is utilized, limiting the number of reagent reservoirs to ten. By extending the design to incorporate the remaining three-quarters of the disc or by providing it with a three-dimensional structure [24], it will be possible to increase the number of titration steps, thereby increasing both the resolution and the range of titration.

Second, improvements in the sealing and reusability of the microfluidic chip are needed. Currently, the reservoirs are manually sealed with adhesive tape, but for better long-term airtightness and scalability, more robust sealing methods—such as thermal bonding or UV-curing adhesives—should be considered. Additionally, the use of more chemically resistant and durable materials should be explored to enhance the structural integrity of the chip.

Third, in terms of future developments, the modular architecture of the system facilitates the integration of additional sensors (e.g. conductivity sensors and ion-selective electrodes), enabling multiparameter simultaneous measurements and compatibility with multistep or sequential reactions, based on its reservoir architecture and programmable rotational control, allowing for timed reagent delivery and independent sensor integration. Furthermore, the portability offered by Bluetooth communication and battery operation renders the system well suited for offsite applications such as ocean monitoring, environmental water testing, and point-of-care diagnostics.

In summary, with continued refinement, the proposed system holds notable potential as a practical alternative to conventional titration methods, offering new opportunities for decentralized and field-deployable chemical analysis.

## Conclusions

This study demonstrated the successful integration of a CD-type microfluidic chip, an ISFET pH sensor, and open-source electronics to develop a compact, automated titration system capable of operating under microlitre-scale conditions. The system enabled real-time pH monitoring and stepwise acid addition without the need for external pumps or complex tubing.

Quantitative evaluation confirmed that the device produced acid–base titration profiles that were consistent with those obtained via conventional glass electrode-based methods, even when the sample volume was reduced to 1/30 of the original volume. Despite its low-cost and portable design, the system exhibited sufficient measurement accuracy and reproducibility to support its use in analytical chemistry.

Although certain limitations remain, including the discrete nature of titrant delivery and the need for more robust sealing techniques, these issues can be addressed through future improvements in chip design and fabrication. Moreover, the modular nature of the platform allows for future integration of multistep assays, multisensor configurations, and field-ready applications.

Overall, the proposed device offers a practical and flexible alternative to conventional titration instruments, with promising applications in environmental monitoring, marine chemistry, and portable diagnostics. By overcoming key barriers to miniaturization and automation, this work contributes to the advancement of decentralized analytical technologies and lays a foundation for the future development of autonomous, multiple-analyte sensing platforms for resource-limited settings.

## Data Availability

No datasets were generated or analysed during the current study.

## Code availability

The code used in this study is not publicly available but can be provided by the authors upon reasonable request.
